# K^+^ and Ca^2+^ Channels Regulate Ca^2+^ Signaling in Chondrocytes: An Illustrated Review

**DOI:** 10.3390/cells9071577

**Published:** 2020-06-29

**Authors:** Yoshiaki Suzuki, Hisao Yamamura, Yuji Imaizumi, Robert B. Clark, Wayne R. Giles

**Affiliations:** 1 Department of Molecular & Cellular Pharmacology, Graduate School of Pharmaceutical Sciences, Nagoya City University, 3-1 Tanabedori, Mizuhoku, Nagoya 467-8603, Japan; yamamura@phar.nagoya-cu.ac.jp (H.Y.); yimaizum@phar.nagoya-cu.ac.jp (Y.I.); 2 Department of Physiology and Pharmacology, Cumming School of Medicine, University of Calgary, 2500 University Drive NW, Calgary, AB T2N 1N4, Canada; rclar@ucalgary.ca (R.B.C.); wgiles@ucalgary.ca (W.R.G.)

**Keywords:** chondrocyte, OUMS-27, resting membrane potential, Ca^2+^ signaling, Ca^2+^ release-activated Ca^2+^ channel, Ca^2+^-activated K^+^ channel, total internal reflection fluorescence microscopy

## Abstract

An improved understanding of fundamental physiological principles and progressive pathophysiological processes in human articular joints (e.g., shoulders, knees, elbows) requires detailed investigations of two principal cell types: synovial fibroblasts and chondrocytes. Our studies, done in the past 8–10 years, have used electrophysiological, Ca^2+^ imaging, single molecule monitoring, immunocytochemical, and molecular methods to investigate regulation of the resting membrane potential (E_R_) and intracellular Ca^2+^ levels in human chondrocytes maintained in 2-D culture. Insights from these published papers are as follows: (1) Chondrocyte preparations express a number of different ion channels that can regulate their E_R_. (2) Understanding the basis for E_R_ requires knowledge of (a) the presence or absence of ligand (ATP/histamine) stimulation and (b) the extraordinary ionic composition and ionic strength of synovial fluid. (3) In our chondrocyte preparations, at least two types of Ca^2+^-activated K^+^ channels are expressed and can significantly hyperpolarize E_R_. (4) Accounting for changes in E_R_ can provide insights into the functional roles of the ligand-dependent Ca^2+^ influx through store-operated Ca^2+^ channels. Some of the findings are illustrated in this review. Our summary diagram suggests that, in chondrocytes, the K^+^ and Ca^2+^ channels are linked in a positive feedback loop that can augment Ca^2+^ influx and therefore regulate lubricant and cytokine secretion and gene transcription.

## 1. Introduction

Advancing present understanding of mechanisms that underlie physiological responses in musculoskeletal systems requires detailed measurements and analyses made in both the active or excitable cell types (e.g., skeletal muscle fibers, neurons) and the closely associated non-excitable cells (chondrocytes, fibroblasts, glia, endothelial cells) that make up these tissues and organs [1]. In articular joints (elbows, shoulders, knees), at least two types of non-excitable cells need to be studied: synovial fibroblasts and chondrocytes. In healthy articular joints, both of these cells synthesize and secrete essential lubricants [2] (hyaluronan, lubricin) and both also exhibit altered function associated with progressive development of rheumatoid and/or osteoarthritis [3,4,5,6,7,8] and related pain [9].

It is well known that the precise regulation of intracellular Ca^2+^ levels ([Ca^2+^]_i_) is required for normal matrix metabolic and secretory functions, as well as cartilage differentiation [10,11]. Dysregulation of [Ca^2+^]_i_ homeostasis is very likely to be an important factor in the initiation and rate of progression of chronic articular joint diseases such as arthritis [3,6,7,8] and related inflammation [12].

In our published studies that form the basis of this review we have combined cellular electrophysiology, whole cell [Ca^2+^]_i_ monitoring and spatial imaging, as well as immunocytochemical and molecular biology methods to identify and define some of the dynamic regulatory mechanisms for [Ca^2+^]_i_ in chondrocytes. In this summary, our main goal was to define functional relationships between K^+^ channel regulation of the resting membrane potential (E_R_); and [Ca^2+^]_i_ levels, both at baseline and following the application of physiological ligands. In addition, selected (previously published) data sets, based on total internal reflection fluorescence (TIRF) microscopy, provide a basis for relating changes in cellular or macroscopic properties to ligand-induced intracellular trafficking of defined components of ion channel complexes such as those for Ca^2+^-activated K^+^ (K_Ca_) channels [13] and store-operated Ca^2+^ (SOC) channels also denoted Ca^2+^ release-activated Ca^2+^ (CRAC) channels [14]. Our published findings on human chondrocytes and chondrocyte cell lines, combined with data from recent literature, form the basis of this illustrated review. 

## 2. Materials and Methods

**Figure 1:**
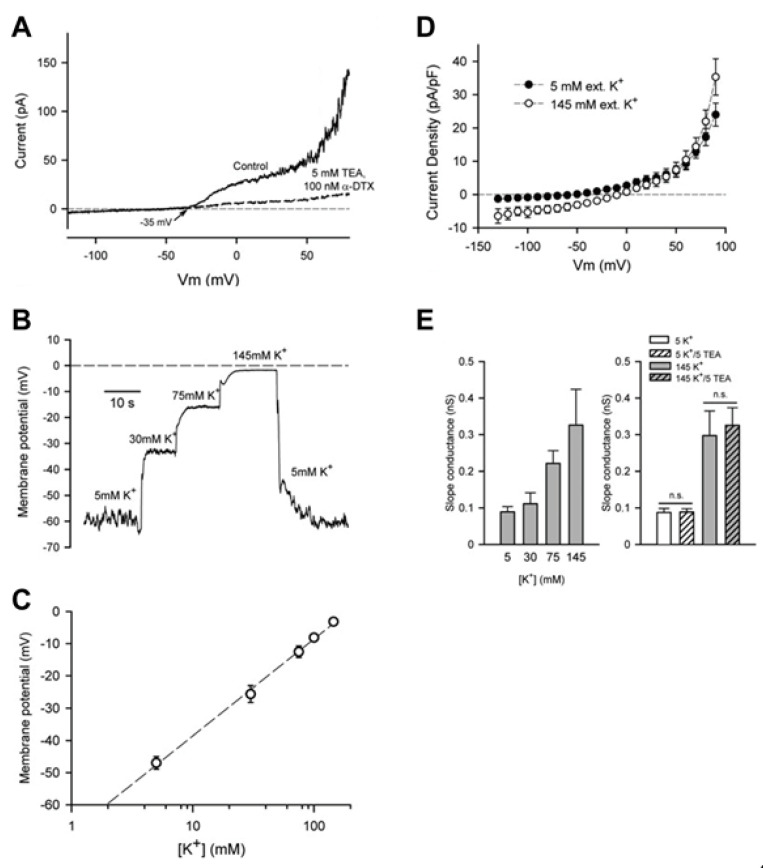
Resting membrane potential and K^+^ currents in an isolated human articular chondrocyte. (**A**) shows the changes in outward currents in response to simultaneous application of 5 mM TEA plus 100 nM dendrotoxin. These compounds block Ca^2+^-activated K^+^ channels and time- and voltage-dependent K^+^ channels respectively. The experimental records in (**B**), and the summarized data in (**C**) show that the membrane potential in these isolated human chondrocytes that were maintained in 2-D cell culture is strongly dependent upon the electrochemical gradient for K^+^. Panels (**A**), (**B**) and (**C**) are taken from our published paper [15]. The experimental data in (**D**) and summarized data sets of results in (**E**) reveal an additional, small but functionally important, time-independent K^+^ current in the range of membrane potentials approximately −40 to −100 mV. In these experiments each chondrocyte (*n* = 7) was first exposed to 5 mM [K^+^]_o_, and then to isotonic [K^+^]_o_ in an effort to reveal small background current as described in detail in Figure 6 of the corresponding published paper [15]. In this paper we conclude that the current change shown in Panel D is produced by 2-pore K^+^ channels.

**Figure 2:**
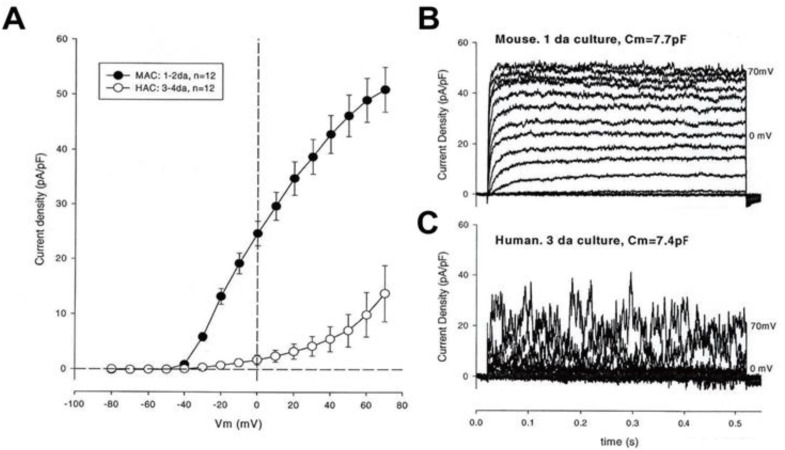
Qualitative comparison of transmembrane K^+^ currents recorded from single isolated murine and human articular chondrocytes that were maintained in primary culture. The two superimposed families of currents in (**B**) and (**C**) on the right show marked differences in quantitative features between recordings from mouse (MAC) vs. those from humans (HAC). The two superimposed current voltage curves in (**A**) of this Figure further illustrate these differences (Clark and Giles, unpublished). Our previously published papers have revealed quite marked differences in the relative functional expression of K^+^ currents when murine [16], canine [20] and human [15] data sets are compared. Detailed methods can be also found in these previously published articles.

**Figure 3:**
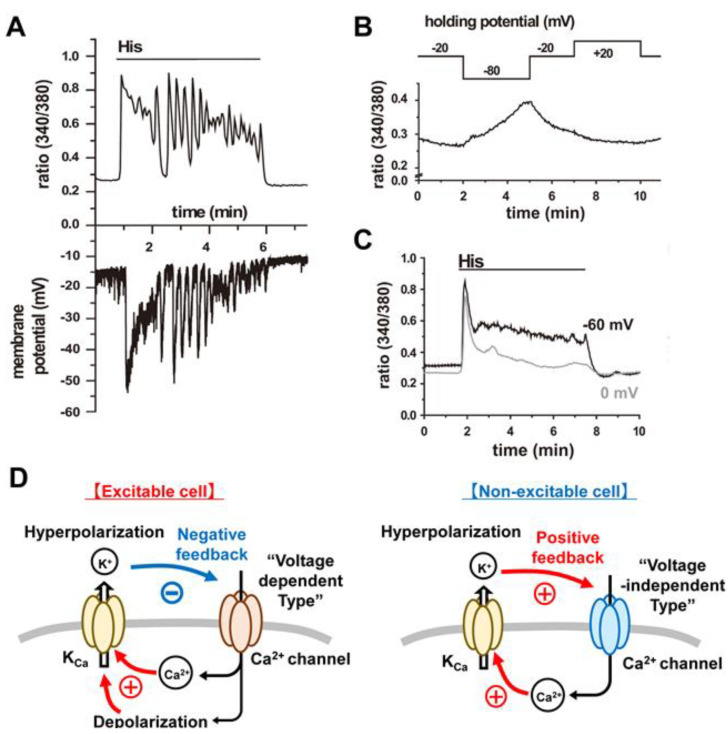
Relationship between change in membrane potential and [Ca^2+^]_i_. (**A**) Whole cell patch techniques were applied to OUMS-27 cells loaded with fura-2 from recording pipette. Typical simultaneous recording of [Ca^2+^]_i_ signals (top) and membrane potential (bottom) under current-clamp mode. (**B**) [Ca^2+^]_i_ signals were measured during voltage-clamp protocols in the absence of 1 µM histamine. (**C**) the black and gray lines indicate changes in [Ca^2+^]_i_ measured in the presence of 1 µM histamine at holding potential of −60 and 0 mV, respectively. These data clearly showed that membrane hyperpolarization increases Ca^2+^ influx through voltage-independent Ca^2+^ channel (VICC). Figure 3A–C were obtained from Ref [17]. (**D**) diagram of relationship between change in membrane potential and [Ca^2+^]_i_ in excitable and non-excitable cells. (Left) In excitable cells, depolarization opens voltage-dependent Ca^2+^ channels (VDCC), which causes Ca^2+^ influx as well as further depolarization. In these cells, K_Ca_ channel, especially BK_Ca_ channel, is activated, and subsequently causes hyperpolarization. This hyperpolarization decreases Ca^2+^ influx through VDCC. Thus, BK_Ca_ channel functions as negative feedback to VDCC activity. (Right) In non-excitable cells, VICC, not VDCC, is main Ca^2+^ channel. CRAC channel is a representative VICC and activates K_Ca_ channel, which results in hyperpolarization. The gating of CRAC channel is not closed by hyperpolarization because CRAC channel does not have voltage-sensing domain. As long as STIM opens the pore of CRAC channel, CRAC can conduct Ca^2+^ even at the hyperpolarized potentials. Hyperpolarization caused by K_Ca_ channel increases driving force for Ca^2+^ and thus promotes Ca^2+^ influx through CRAC channel. Therefore, BK_Ca_ channels contribute to positive feedback for Ca^2+^ influx through CRAC channels. Note that CRAC channel activation causes very small membrane depolarization because their single channel conductance (9 fS [29]) is much smaller than that of VDCC (L-type Cav1.2 channel: 2.4 pS [30]). Therefore, quite small depolarization by Ca^2+^ influx through CRAC channels is lost because of large membrane hyperpolarization caused by K^+^ conductance through BK_Ca_ channel (200 pS [19]).

**Figure 4:**
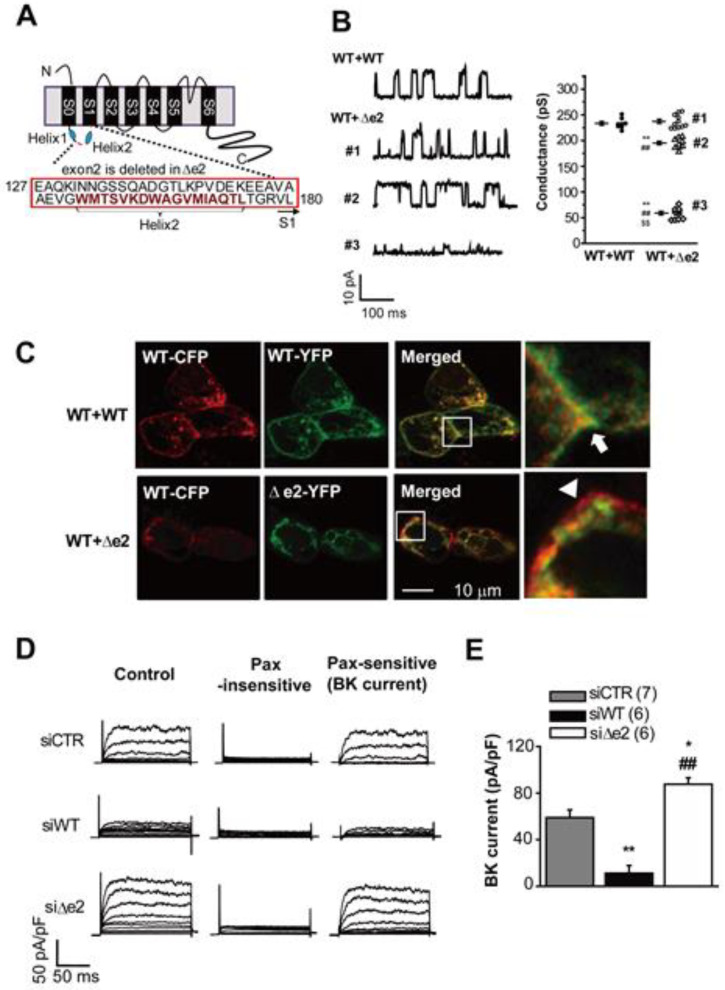
(**A**) Schematic diagram of BKαΔe2. This splice variant lacks exon2, which codes approximately half of S0-S1 linker (C-terminal side) and first two residues (^179^VL^180^) in the S1 segment (**B**) Single channel currents (left) were obtained at +50 mV from HEK cells expressing both control or wild type (BKαWT) and splice variant (Δe2). In control cells (WT+WT), these unitary currents show a single amplitude distribution pattern with a peak at 11.2 pA. In contrast, the amplitude histogram in WT+Δe2 includes three distinct groups of single channel amplitudes (#1, 11.1 pA; #2, 9.7 pA; #3, 3.5 pA). (Right) Summary of single channel conductances in WT+WT and WT+Δe2. **, *p* < 0.01 vs. WT+WT; ##, *p* < 0.01 vs. #1; $$, *p* < 0.01 vs. #2. (**C**) Confocal images of cells co-expressing WT-CFP+WT-YFP (top row), or WT-CFP+∆e2-YFP (lower row). Note that in merged and expanded areas (enclosed by squares) in WT+WT, significant co-localization (yellow) at the plasma membrane (PM) is observed as indicated by the arrow. In contrast, the WT+Δe2, CFP fluorescence signal (red) indicates no co-localization at the PM as shown by the arrow. (**D**) Outward currents recorded in OUMS-27 cells treated with siRNA specifically targeting WT or Δe2. Currents recorded before (left) and after (middle) the application of 1 μM paxilline (Pax, a selective BK_Ca_ channel blocker) are shown along with Pax-sensitive currents (BK_Ca_ current, right). (**E**) Comparisons of these current densities at +120 mV. *, *p* < 0.05; **, *p* < 0.01 vs. siCTR; ##, *p* < 0.01 vs. siWT. This research was originally published in the *J. Biol. Chem.* 2016; 291:24247–24260. © the American Society for Biochemistry and Molecular Biology (see [19]).

**Figure 5:**
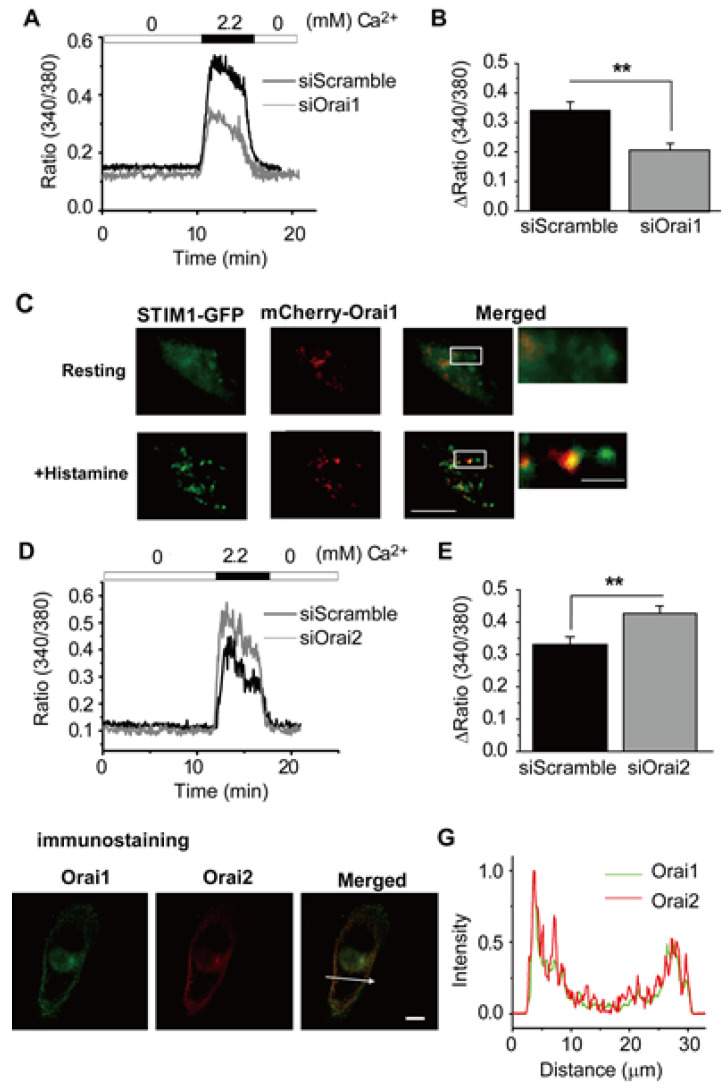
(**A**) shows [Ca^2+^]_i_ changes following ER store depletion due to addition of 2 μM thapsigargin to OUMS-27 cells that had been transfected with either siOrai1 or siScramble. (**B**) summarizes the data based on the Ca^2+^ signal (** *p* <0.01). (**C**) Molecular dynamics based on Orai1 and STIM1 interactions were visualized under physiological conditions. Note that application of 10 μM histamine-induced punctate STIM1–Orai1 complex formation. Scale bars denote 5 μm (left) and 1 µm (right), respectively. (**D**) Compares SOCE in cells transfected with siOrai2 or siScramble. The corresponding [Ca^2+^]_i_ signals compared in (**E**) demonstrates statistical significance (** *p* < 0.01). (**F**) Shows double immunostaining images of Orai1 (green) and Orai2 (red) in OUMS-27 cells. The scale bar denotes 10 μm. (**G**) shows the relative intensity profile corresponding to the location of the arrow in Panel F. Note also that (yellow region in Panel F) Orai1 and Orai2 showed a similar distribution pattern (see [18]). Reprinted from *Cell Calcium*, 57, Inayama M et al., Orai1–Orai2 complex is involved in store-operated calcium entry in chondrocyte cell lines, 337–347, Copyright (2015), with permission from Elsevier (see [18]).

The methods and the data sets that we have used to generate Figure 1, Figure 2, Figure 3, Figure 4 and Figure 5 have been described in detail in our previous publications. Specifically, the whole cell patch clamp methods [15], cell isolation and culture techniques [16,17] and optical routines for macroscopic recordings of ligand-induced changes in [Ca^2+^]_i_ [17,18] have been published previously. Details concerning molecular biology methods and those for spatially resolved imaging of Ca^2+^ or translocation of intracellular protein substituents using TIRF microscopy can be found in [18,19]. We have also published a mathematical model illustrating the roles of K^+^ channels in the generation of the chondrocyte E_R_ [20,21].

## 3. Results

### 3.1. Chondrocyte Resting Membrane Potential

The chondrocyte is a non-excitable cell, and yet it expresses a wide variety of ion channels, pumps and exchangers [22,23,24,25]. Gaining an understanding for the basis of its E_R_ therefore is of strong interest. Our groups [15,16,17,18,19,20,21] and others [26] have addressed this problem using both experimental and mathematical modeling approaches.

Soon after initiating this project in 2008, we realized that valid determination of the E_R_ in any cell type having the biophysical characteristics of an adult human chondrocyte requires a clear appreciation of the technical limits that are always associated with application of the conventional patch clamp technique (that is used for recording transmembrane ionic currents) [20]. In brief, the very small size (approx. 6 pF) and the commensurate, very high input resistance (approx. 10 GΩ) that are characteristic of a healthy human chondrocyte require optimization of the patch clamp electrode so that consistent and very high seal resistances can be obtained (and documented) for each successful recording. In the absence of this, the ‘seal leakage current’ can contaminate or even dominate the true or intrinsic E_R_ value [20]. In our experience, leakage current through the seal resistance often results in recording a stable potential of perhaps -20 mV, as opposed to a value between –40 and –45 mV that is likely to be more representative but perhaps also not completely correct (see Discussion).

Almost all of our studies of the chondrocyte E_R_ have been made under experimental conditions (ion substitution and/or addition of, e.g., Cl^−^ channel blockers) in which the presence and the physiological roles of baseline or control currents that are mediated by K^+^ channels were the main focus [15,16]. Examples of two of our published data sets recorded from single isolated human articular chondrocytes have been combined and are shown in Figure 1. Panel A illustrates the marked reduction when K^+^ currents are blocked using a combination of tetraethylammonium (TEA), and dendrotoxin. TEA is an effective but somewhat non-selective blocker of the large conductance Ca^2+^-activated K^+^ (BK_Ca_) current, and dendrotoxin is a blocker of time- and voltage-dependent delayed rectifier K^+^ current. The experimental records in Panel B and the summary of this data in Panel C show that the E_R_ can be strongly regulated by the electrochemical gradient for K^+^. However, careful inspection of this published data also reveals that increases in [K^+^]_o_, in fact, produce only very small changes in transmembrane current in the region of membrane potentials close to the E_R_. We have explored this in detail [15] and some of our findings are shown in Panels D and E of Figure 1. The current-voltage relationship in Panel D and the summarized data in Panel E reveal that a different, TEA and dendrotoxin-insensitive background or time-independent current can be identified in the range of membrane potentials (−50 to −80 mV) where the E_R_ would be expected. In brief, the data in Figure 1 and other published studies strongly suggest an important role for distinct subtypes of the 2-pore K^+^ channel family in generating the resting potential in an unstimulated chondrocyte [15].

However, in some human chondrocytes and in the majority of chondrocytes from the adult mouse [16] and canine [20] knee joint, a time- and voltage-dependent delayed rectifier K^+^ current is also strongly expressed and is likely to play a significant functional role. Our preliminary findings regarding the functional expression of this K^+^ current and the quite marked differences in its expression density, mouse vs. human, are illustrated in Figure 2. In addition, all chondrocytes from the three different mammalian preparations that we have studied, as well as a chondrocyte cell line (OUMS-27) in primary culture, express Ca^2+^-activated K^+^ (K_Ca_) conductances of at least two types: the large conductance [13], and the intermediate conductance [27]. When activated, either or both of these ligand-gated transmembrane K^+^ currents not only modulate but in fact can dominate the chondrocyte membrane potential. It can be driven in the hyperpolarizing direction to perhaps −80 mV very near the electrochemical equilibrium potential for K^+^ in most physiological conditions (see [2,28] and Discussion). 

### 3.2. Large Conductance Ca^2+^-Activated K^+^ (BK_Ca_) Channels

The exceptionally broad expression profile and well-established physiological roles for the BK_Ca_ channels [13] motivated us to study this current in both adult chondrocytes and an available chondrocyte-like cell line, OUMS-27. In both of these chondrocyte preparations [15,16,17,18,19] our molecular, immunocytochemical, electrophysiological, and Ca^2+^ imaging measurements revealed that activation of the BK_Ca_ current by bath application of histamine, or by controlled increases in [Ca^2+^]_i_, consistently resulted in a significant hyperpolarization of the E_R_ (Figure 3A). This primary effect had the important secondary consequence of increasing the electrochemical driving force and therefore augmenting Ca^2+^ influx through voltage-independent, Ca^2+^ selective, channels such as those mediated by the transient receptor potential (TRP) channel or CRAC channels (Figure 3B,C). In chondrocytes, as in many other non-excitable cell types this increase in [Ca^2+^]_i_ acts as a potent stimulus for BK_Ca_ channel and the further, (and usually much larger and longer) Ca^2+^ influx as well as release of Ca^2+^ from intracellular stores within the endoplasmic reticulum (ER) (see Section 3.3 and Section 3.4).

Interestingly, when studying the roles of BK_Ca_ channels in chondrocytes we also detected the presence of splice variants of the α subunit of the BK_Ca_ channel protein complex in a chondrocyte cell line, OUMS-27 [19]. One of the splice variants, denoted ∆e2 that we identified (Figure 4A), strongly modulates key aspects of the native BK_Ca_ channels: it reduces single channel conductance, decreases BK_Ca_ expression levels in the surface membrane and also alters the voltage-dependence for activation of the native BK_Ca_ current [19]. Interestingly, homotetramers of BKα∆e2 and heterotetramers of BKαWT+BKα∆e2 are localized in intracellular compartments, although homotetramers of BKαWT preferentially move to plasma membrane (See Figure 4D). The deleted region in BKα∆e2 are predicted to have an ER export signal, so the possibility that BK_Ca_ channel containing BKα∆e2 may be localized in ER and function as intracellular organellar channels cannot be excluded. Furthermore, BK_Ca_ channel activity is regulated by membrane lipid [31,32,33]. Since BK_Ca_ channels with or without BKα∆e2 are located in distinct membranes, i.e., cell surface and intracellular organelles, they may be modulated differentially by membrane lipids specific to each organellar membranes. 

We speculate that this type of genetically-induced heterogeneity may function in the setting of articular joint disease [3,4,5]. Specifically, this splice variant-induced decrease in BK_Ca_ current attenuated histamine-induced cyclooxygenase (COX) 2 gene transcription that is controlled by Ca^2+^-dependent factors, such as NFAT [34] and NF-κB [35], an effect that may also alter the lubricant secretion by the chondrocyte and its ability to proliferate in settings such as osteoarthritis or rheumatoid arthritis (see Discussion).

### 3.3. Intermediate Conductance Ca^2+^-Activated K^+^ (IK_Ca_ or K_Ca_3.1) Channels: 

Our investigations of the electrophysiological and biophysical properties of isolated human chondrocytes have also suggested that there are significant effects of the activation of Ca^2+^-activated K^+^ channels of the intermediate conductance subtype. These are denoted, IK_Ca_ or K_Ca_3.1 (for reviews, see [27,36,37]). Based on our preliminary results (Clark and Giles, unpublished) and our published studies of this conductance in human synovial fibroblasts [2,38], this K^+^ current is normally very small and sometimes not detectable at baseline (in the unstimulated cell) in both of the principal cell types of articular joints. However, it can be augmented substantially by physiological levels of ATP or ADP and also by endocrine or paracrine substances that are known to be present in synovial fluid in both healthy and progressive disease settings [39,40,41]. When it is activated, this K^+^ current dominates those that otherwise regulate the E_R_ in the chondrocyte [28] or synovial fibroblast [2,38], Accordingly, this K^+^ current drives the membrane potential in the hyperpolarizing direction and then holds it there for relatively long times (2–10 sec), effects that depend strongly upon the specifics and the lifetime of the ligand agonist. The relatively large hyperpolarization (e.g., from −40 mV to −75 mV) increases the driving force for Ca^2+^ and Na^+^ and thereby augments mainly Ca^2+^ influx in a fashion quite similar to that described in Section 3.2 above for BK_Ca_ channels/currents. Overall, this scheme strongly resembles what is quite well established concerning the functional roles for IK_Ca_ in defined cell types in the immune system [42,43]. These insights are the basis for ongoing, active searches for IK_Ca_ targeted approaches to alter immune function [44] based in part on alterations of [Ca^2+^]_i_ signaling in lymphocytes and T-cells [45].

### 3.4. Ca^2+^ Release-Activated Ca^2+^ (CRAC) Channels

In a large number of quite different non-excitable cells, an essential component in the overall [Ca^2+^]_i_ homeostatic response involves voltage-independent Ca^2+^ channel-mediated communication between the Ca^2+^ influx across the surface membrane of the cell and signaling that is dependent upon the Ca^2+^ content in intracellular organelles (mainly the ER) [14,46]. This type of store operated Ca^2+^ entry, denoted SOCE:

i. Can be initiated by a wide variety of signals at the surface membrane (e.g., ATP activation of purinergic receptor subtypes, or stretch) that result in a small net influx of Ca^2+^ and/or of Ca^2+^ and Na^+^.
ii. Almost always requires activation of submembrane phospholipase C as the first step in an intracellular signaling pathway that produces ultimately IP_3_. IP_3_ is a potent second messenger that activates significant Ca^2+^ release from the ER [47].
iii. Is characterized by movement of an ER localized Ca^2+^ sensor protein (Stromal Interaction Molecule (STIM) 1–2) to discrete junctions of the ER/plasma membrane [48].
iv. Is completed by functional association of these STIM proteins with a second, distinct class of proteins (Orai1–3) at the ER/plasma membrane junctions, resulting in the formation of Ca^2+^ selective channels [49,50]. These hybrid channels are responsible for the maintained Ca^2+^ influx and related non-inactivating inward current, denoted I_CRAC_ or I_SOCE_ [50].

This dynamic process is initiated and then regulated by the depletion of [Ca^2+^]_i_ in sub-compartments of the ER. Very shortly after its activation, the functional phenotype of the parent cell (e.g., the chondrocyte) changes significantly:

i. There is a marked hyperpolarization of plasma membrane due to activation of one or more of the subtypes of K_Ca_ channels that are expressed.
ii. The chondrocyte responds to the change in [Ca^2+^]_i_, by sometimes generating [Ca^2+^]_i_ waves or oscillations [51]. These can activate cascades of Ca^2+^-dependent enzymes, (CaMK2 [6,7], NFAT, and calcineurin [8,11]); enhanced secretion of cytokines, catabolic factors and paracrine substances [52,53]; increased Ca^2+^-dependent secretion of essential extracellular matrix ([2,10,38,54]) and significant changes in cellular transcription activity [10,54] and/or altered proliferation as well as differentiation [11,51,55].

Based on the assumption that it is likely that chondrocyte function also is regulated by store operated Ca^2+^ fluxes, we undertook a detailed study of this in the chondrocyte cell line OUMS-27 [18]. Our results (Figure 5) revealed that CRAC channel-mediated Ca^2+^ influx (Orai1, Orai2 and STIM1) contribute significantly to the regulation of [Ca^2+^]_i_ levels both in the resting and histamine-activated state. In addition, in the OUMS-27 chondrocyte-like cell line [18], we found that Orai1 and Orai2 can form heteromeric complex to constitute CRAC channels in OUMS-27 cells although homomers of Orai1 or Orai2 can make functional CRAC channels. Orai2 can significantly regulate Orai1 activity by forming heteromers, thereby altering the Ca^2+^ currents carried by CRAC channels. The detailed mechanism of this inhibitory effect is unclear, but some differences in amino acid sequence between Orai1 and Orai2 may influence interaction between Orai and STIM1 and gating properties such as Ca^2+^ dependent inactivation. Some of these findings are illustrated in Figure 5. The involvement of this, or very similar intracellular signaling pathways, in essential aspects of innate and/or adaptive immune responses [43] has led to the channel proteins that are responsible for I_CRAC_ being actively explored as drug targets in the setting of, e.g., autoimmune diseases [56]. 

## 4. Discussion

### 4.1. Main Findings

One of the main insights from this illustrated review is that, in the adult human chondrocyte (and at least one cell line, OUMS-27, a model for chondrocyte cell physiology), a significant regulatory paradigm for [Ca^2+^]_i_ homeostasis and definition of cell phenotype can be described as a feedback loop involving:

i. Ligand (ATP or histamine) triggered release of Ca^2+^ from one or more intracellular stores (e.g., ER).
ii. Targeted translocation of a specific intracellular protein (STIM1) from the ER to discrete spatial locations near the surface membrane (the ER-plasma membrane junction).
iii. STIM1-induced conformational changes in a second partner protein (Orai1) and resulting formation of ion channels that readily allow Ca^2+^ to enter the chondrocyte over quite extended (s) time periods. This is referred to as SOCE, and takes place through CRAC channels.
iv. Alterations (hyperpolarization) in chondrocyte E_R_ triggered by increases in [Ca^2+^]_i_ and resulting augmentation of Ca^2+^ influx then initiates or promotes functionally important enzymatic cascades or intracellular regulatory pathways (Ca^2+^-dependent phosphorylation/dephosphorylation).

A key component in this scheme is the ligand-triggered, dynamic formation of CRAC channels and subsequent maintained influx of Ca^2+^. However, transmembrane current corresponding to this influx can be challenging to record directly, or to identify with certainty. This is due to its relatively small size and the need for additional selective, reversible compounds that can block these channels. Our approach made use of siRNA and a dominant negative form of Orai1 (Orai1 [E106Q]) clearly demonstrated functional expression of CRAC channels in chondrocytes. 

### 4.2. Relationship to Previous Reports of Other Ion Channels and Transporters in Adult Chondrocytes

#### 4.2.1. TRP Channels and Piezo Stretch Sensitive Channels

Previous molecular and electrophysiological studies, as well as assays for changes in [Ca^2+^]_i_ and gene expression, have consistently revealed expression and function of a small number of TRP channel transcripts in chondrocytes [24,57]. Among these, TRPV4 is perhaps the most predominant [55]. Activation of TRPV4 channels results in brief but significant Ca^2+^ influx into chondrocyte preparations [54,58,59,60]. TRPV4 channels have been studied in detail to establish the basis for their sensitivity to changes in osmotic strength [58,59] and dynamic loading [54]. 

Some of the same investigators that have studied TRP channel function in mammalian chondrocytes have also recently reported results revealing the co-expression of the stretch-sensitive or ‘stretch-transducer ion channel’ [60,61,62]. These have been named Piezo channels [63]. Both Piezo-1 and Piezo-2 are functionally expressed in a number of different adult mammalian chondrocyte preparations [64]. 

Given the fact that chondrocytes in articular joints experience cyclical compression and decompression, and perhaps also stretch and shear, it is likely that physiological activation of such Piezo channels plays an important role in normal physiological function [61]. If so, activity-dependent bursts of Ca^2+^ influx [62] will constitute an important early step in signal transduction. Interestingly, TRPV4 and Piezo channels exhibit different thresholds for activation by mechanical stimuli. TRPV4 detects even very weak mechanical perturbations while Piezo channels are activated by stronger stimuli. It is plausible, therefore, that TRPV4 may be a primary physiological mechano-sensor; while Piezo channels detect stronger or more noxious stimuli [60,62,64], possibly implying that they have different functional roles in healthy vs. disease settings [61].

The magnitude and perhaps the gain of this response would be expected to be dependent upon the details of the incident, repetitive mechanical activity. We suggest that it will also be modulated by the electrochemical driving forces for Ca^2+^ and hence be strongly regulated by the exact value of the chondrocyte E_R_. It is also known that this initial Ca^2+^ influx may give rise to maintained [Ca^2+^]_i_ oscillations or intracellular waves [51,65]. This is sometimes referred to as ‘mechanoregulation’ and this [Ca^2+^]_i_-driven signaling can markedly enhance gene expression [54,66] as well as chondrocyte proliferation and maturity [67]. 

#### 4.2.2. Ca^2+^ Channels in Mammalian Chondrocytes

Published results, based mainly on molecular analyses and gene expression profiles of human chondrocyte lysates, quite consistently provide evidence for functional Ca^2+^ channels in these cells [24]. Indeed, there are detailed descriptions of both T- and L-type Ca^2+^ channel function on mouse chondrocytes [68,69]. In our experience, based mainly on studies of canine and human chondrocytes, however, consistent recording of L-type Ca^2+^ channel activity from chondrocytes placed in primary culture has not been possible. This is true even under conditions when the E_R_ is strongly hyperpolarized (e.g., from −40 to −80 mV) and hence the electrochemical driving force for both Ca^2+^ is relatively large. Although the reasons for our negative results are not known, it is likely that our experimental conditions are not favourable. Our experiments were performed at room temperature (23 °C) as opposed to physiological body temperature (37 °C), and our pipette filling solution that dialyses the chondrocyte cytosol did not include the high-energy phosphate compounds that are known to enhance and stabilize L-type Ca^2+^ currents in other mammalian cells. It is also possible that our inability to record Ca^2+^ currents is due in part to a lack of required stimulating ligands (e.g., histamine or ATP) in our superfusate solution. Additional study of Ca^2+^ channel expression and function in human chondrocytes is much needed, but is likely to continue to present technical challenges for investigators that use conventional patch clamp methods.

#### 4.2.3. Na^+^/K^+^ Pump Expression in Mammalian Chondrocytes

Evidence supporting functional expression of a number of different isoforms of both the α and β subunits of the Na^+^/K^+^ pump protein complex was first published more than 10 years ago [70,71]. Our preliminary gene expression studies (Belke et al., unpublished) done on human chondrocytes support these important previous immunocytochemical findings [72]. 

There are at least three reasons for cell physiologists having strong interest in Na^+^/K^+^ pump expression and function in chondrocytes under both physiological and pathophysiological conditions.

i. In chondrocytes and in perhaps all other cells the Na^+^/K^+^ pump plays a primary role in setting and regulating cell volume [23,26,73].
ii. The immediate environment of the chondrocyte, the synovial fluid, has an ionic composition that would be expected to strongly stimulate Na^+^/K^+^ pump turnover under physiological conditions [21], given its molecular composition (the α2 subunit of the Na^+^/K^+^ pump [74]).
iii. The net outward current generated by Na^+^/K^+^ pump turnover, although small (perhaps 10 pA) is capable of hyperpolarizing the membrane potential of the chondrocyte by 10–15 mV, as we have shown using mathematical modeling approaches [21].

#### 4.2.4. Connexin and Pannexin-Based Channels and Signaling

The expression of connexin proteins in chondrocyte preparations from adult donors may have been a somewhat puzzling finding initially. This is because with the exception of chondrocytes in the growth plate, these cells are understood to function as ‘stand alone’ or single isolated entities. However, data demonstrating that connexins, when expressed in surface membranes of a variety of adult mammalian cells, can function as ‘hemi channels’ has placed the previously observed expression of connexins in a novel and interesting context [75]. For example, Knight and his colleagues [76] have reported that specific connexin family members (e.g., connexin 43) act in concert with specific members of the purinergic receptor family to form a functional mechano-sensitive unit in adult chondrocytes. This signaling complex is very sensitive to stretch (and perhaps shear forces) imposed on the chondrocyte and reacts with significant release of ATP through the relatively large connexin 43 hemi channel pore [77]. The extracellular ATP then binds to immediately adjacent purinergic receptors resulting in potent, and both spatially and pharmacologically selective, activation of the chondrocyte. Interesting results from additional studies have raised the possibility that this signaling pathway, again involving [Ca^2+^]_i_ oscillations can also coordinate chondrocyte migration with the level of repetitive mechanical activity of the articular joint [78]. 

Related multidisciplinary research that has also significantly advanced present understanding of chondrocyte cell physiology, mechanobiology, and pathology is based upon the discovery of expression and functional activity of additional connexin-like proteins. This is the pannexin family, (specifically 1 and 3), specific members of which have been identified in chondrocyte surface membranes [79]. Recent work from a number of groups has documented this functional expression and revealed that pannexin hemi-channels provide a second and significant pathway for stimulus-evoked ATP release from chondrocytes [80,81]. After activating purinergic receptors, this ATP signal results in an increase in intracellular IP_3_ which is a potent activator of Ca^2+^ release from intracellular stores. Since increases in [Ca^2+^]_i_ can activate K_Ca_ channels in chondrocytes the ATP flux through connexin and pannexin-based hemi channels produces a significant hyperpolarization of the E_R_. This results in a marked change in chondrocyte functionality (e.g., secretion, migration) and phenotype (e.g., proliferative) in both healthy and diseased conditions. 

#### 4.2.5. Cl^−^ Channels in Chondrocytes

Although this review and others focus on the functional roles of a number of different K^+^ channel family members in chondrocytes, there is no doubt that Cl^−^ expression can strongly regulate and even modify the E_R_ in both developing and mature chondrocytes. We have provided detailed accounts of an important role for Cl^−^ conductance in regulating ER in the chondrocyte-like cell line OUMS-27 [82,83]. 

Important work that has demonstrated a functional role for Cl^−^ conductance in chondrocytes has been published by the Barrett-Jolley group [84] and others [85,86] in addition to comprehensive gene array surveys, this group has published electrophysiological data sets providing strong evidence for an important role for altered Cl^−^ conductance in the setting of changes in superfusate osmotic strength [23,26]. This context is important since the osmolarity of synovial fluid in the articular joint is definitely in the hyperosmolar range and this parameter is known to change as a function of initiation of inflammation or progressive chronic diseases such as osteoarthritis [87]. 

In most situations, that are relevant to chondrocyte cell physiology the endogenous or baseline Cl^−^ conductance is unlikely to play the predominant role in setting the E_R_ [26]. When considering its functional role, it is important to keep in mind the exact conditions with regard to intra- and extracellular Cl^−^ levels. These factors determine the electrochemical equilibrium potential for Cl^−^. This is typically about −40 mV. Since this is very close to the E_R_ the voltage change produced by the Cl^−^ conductance will be small. However, when the E_R_ is strongly hyperpolarized the Cl^−^ conductance will increase and its influence needs to be considered exactly as it does in the case of Cl^−^ conductances in CNS synaptic physiology and some skeletal muscle cell types.

The functional roles of baseline (healthy) and ligand-activated Cl^−^ conductance in mammalian cells also need to be considered after recognizing that in a number of different mammalian cells intracellular organelles are known to express ion channels including some of those in the Cl^−^ channel family [88]. Cl^−^ channels have been identified in ER, sarcoplasmic reticulum (SR) and intracellular vesicles such as secretory granules [89]. In addition, a number of different K_Ca_ channels (but perhaps mainly the large conductance or BK_Ca_ subtype) are expressed in mitochondria and also in the SR [88]. 

### 4.3. Functional Coupling between CRAC Channels and K_Ca_ Channels: 

#### 4.3.1. An Important Feedback Loop

**Figure 6:**
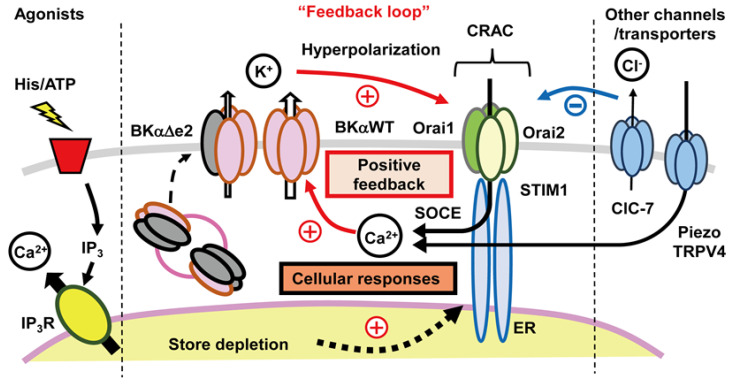
When agonists such as histamine (His) and ATP bind to their receptors, a Gq-mediated phospholipase C enzymatic reaction is triggered. This produces IP_3_ that binds to IP_3_ receptors (IP_3_R) and releases Ca^2+^ from the ER. The resulting Ca^2+^ store depletion causes subsequent SOCE through CRAC channels consisting of Orai1, Orai2 and STIM1. Increases in [Ca^2+^]_i_ activate K_Ca_ channels such as BK_Ca_ and IK_Ca_ channels and cause membrane hyperpolarization. This hyperpolarization further promotes SOCE, thus forming a positive feedback loop that results in a maintained Ca^2+^ influx that can evoke various kinds of cellular responses. Transmembrane Ca^2+^ fluxes are also mediated by other kinds of Ca^2+^-selective channels such as Piezo and TRPV4 channels. These channels sense different types of mechanical stimuli and then transduce them into specific and spatially localized [Ca^2+^]_i_ profiles. Cl^−^ channels/transporters such as ClC-7 are also functionally expressed in a human chondrocyte cell line. ClC-7 depolarizes resting membrane potentials and reduces Ca^2+^ influx through voltage-independent Ca^2+^ channels. In this diagram the splice variant, BKα∆e2, is shown to negatively regulate BK_Ca_ channel activity by forming heterotetramers with BKαWT (see legend of Figure 4).

Published findings mainly concerning the electrophysiological regulation of the innate immune response [42,43,44], have provided a framework for interpreting our work on the linkage between chondrocyte K^+^ channels and ligand-gated activation of SOC channels. This can be done by proposing a robust, high gain feedback loop. This system connects changes in [Ca^2+^]_i_ and the resulting significant, relatively long-lasting hyperpolarization of the E_R_. Figure 6 is a diagram that we have constructed to illustrate some of the established components of this feedback loop, and depicts how the activation of other ligand-gated channels (histamine or ATP) may contribute. 

At least two aspects of this cascade are worth emphasizing. The hyperpolarization of the chondrocyte E_R_ is substantial, (e.g., 20–40 mV), and is often also relatively long lasting (1–10 s). This change in E_R_ can either activate or significantly increase Ca^2+^ influx through both conventional Ca^2+^ channels and CRAC channels. The hyperpolarization also increases the electrochemical driving force for ion fluxes through other channels that are permeable to Ca^2+^ and Na^+^, as well as Cl^−^ channels that are expressed in human chondrocytes [17,82]. In addition, in the chondrocytes that are found in the growth plates of juvenile humans, this hyperpolarization is likely to increase the electrotonic cell-to-cell communication that is mediated by connexin isoforms [80]. The initiation and progression of cell proliferation and/or some types of cell death (apoptosis) as well as cartilage differentiation are also known to be sensitive to both [Ca^2+^]_i_ levels and membrane potential [11,51,54,64]. 

#### 4.3.2. Further Evaluation of Functional Coupling of K^+^ and Ca^2+^ Fluxes

If the scheme shown in Figure 6 is approximately correct, then the chondrocyte E_R_, as a control parameter, is placed squarely ‘in the eye of the storm’. It is a major regulator of the chondrocyte phenotype under physiological conditions. There are many different ways to attempt to further evaluate this working hypothesis. Some of the next steps that we favor are:

i. To use a standard capability of the voltage clamp method in a detailed study of the SOC current, I_CRAC_, activation and dynamics at fixed membrane potentials within the range that a ligand such as ATP produces when it activates K_Ca_ channels.
ii. To evaluate and then implement a novel approach for regulating chondrocyte membrane potential by incorporating optogenetic tools [90], such as synthetic light-sensitive channels including K^+^ channels [91] into chondrocytes in primary culture. This has the advantages of avoiding disruption of the chondrocyte membrane by patch seal formation and allowing repetitive activation of K^+^ channels while also assaying changes in [Ca^2+^]_i_.
iii. To improve throughput of data acquisition using methods that allow ligand-induced changes in chondrocyte E_R_ in populations of isolated cells. It may be possible to monitor and calibrate a signal obtained during flow cytometry assays [92] to provide absolute or near absolute values of the chondrocyte membrane potential. A number of different synthetic or protein-based voltage-sensitive dyes can be evaluated and considered some of which have quite favorable signal-to-noise ratios [90].
iv. To utilize a Systems Biology approach, incorporating an additional set of measurements and calculations. The results would further evaluate the applicability and validity of key Ca^2+^-dependent steps in the diagram shown in Figure 6. Insights from ‘semi quantitative assays’ of [Ca^2+^]_i_ levels at baseline together with parameters describing ligand-induced transients and/or oscillations are much needed. These can be obtained by using recently published analytical software [93]. In other cell and tissue systems, a strong emphasis on details of [Ca^2+^]_i_ transient waveforms has yielded interesting insights into some aspects of [Ca^2+^]_i_ homeostasis [94]. This type of platform-based medium throughput analysis can be put in context and new experiments can be designed by combining these approaches with a mathematical model for ligand-based Ca^2+^ influx, [Ca^2+^]_i_ release and buffering as well as Ca^2+^ extrusion. The Hille group [95] have developed and used this type of rationale and mathematical modeling in their studies of Ca^2+^ homeostasis in the PC-12 cell line.
v. One shortcoming of our working hypothesis, as outlined in Figure 6, is that it does not take full account of the fact that what we denote as distinct ‘ion channels’ almost certainly need to be thought of as ‘ion channel signaling complexes’. This distinction can be illustrated, and interesting new experiments can be planned by re-thinking some key properties of what we have described in this review as the large conductance Ca^2+^-activated K^+^ (BK_Ca_) channel and the intermediate conductance Ca^2+^-activated K^+^ (IK_Ca_) channel.

It is now well known that, even at baseline, BK_Ca_-mediated current is not regulated exclusively by (i) [Ca^2+^]_i_ levels and (ii) transmembrane voltage [13]. Rather, BK_Ca_ channels can also be strongly modulated by ‘endogenous ligands’ such as reactive oxygen species (ROS) or estrogen [96]. It is well known that changes in ROS levels are related to OA [97,98]. When chondrocytes are exposed to various stress such as mechanical stimuli, inflammatory cytokines and hypoxia, ROS including H_2_O_2_, ONOO^−^, NO, CO and H_2_S is generated. Each ROS probably influences wide range of physiological responses of chondrocytes and gets involved in OA pathogenesis. However, the effects of ROS appear to be very complicated: they can show both cytotoxic and protective effects on chondrocytes. It has been reported that ROS modulates BK_Ca_ channel activity by direct or indirect action on BKα or BKβ1 subunits [96]. Therefore, BK_Ca_ channel may partially play roles in ROS-induced effects on chondrocytes during OA progression, but further studies are needed to clarify this point. In addition, the function of BK_Ca_ channels in chondrocytes is almost certainly strongly regulated by the ambient hypoxia of the articular joint. A very important downstream signaling pathway mediated by the hypoxia inducible factor (HIF)-1 family of transcription factors has been shown to significantly alter BK_Ca_ function [99,100]. Hypoxia remains as an important co-factor in the setting of articular joint osteoarthritis [101] and hence BK_Ca_ channel function in this progressive disease may be altered. Interestingly it has recently been shown that BK_Ca_ channel activity can be regulated by circadian activity of the host cell [102]. 

The BK_Ca_ channel has long been known to consist of a signaling complex consisting of the pore or α subunit and an accessory β subunit. Recently, a γ subunit of the BK_Ca_ channel has been cloned and shown to have very strong, indeed profound effects on the voltage-dependence of BK_Ca_ channel and macroscopic current activation [103,104]. Detailed examination for the possibility of γ subunit expression in the human chondrocyte in both health and disease is definitely warranted. 

Somewhat similarly, the functional expression of the intermediate conductance Ca^2+^ channel needs to be considered in the setting of known transcriptional activators [105] and both intrinsic and synthetic relatively selective ‘channel modulators’ [106]. Finally, and perhaps to strongly make the point that neither of these Ca^2+^-sensitive K^+^ channels are likely to act in a stand-alone fashion, it has been shown that when they are co-expressed the level of activity of the intermediate conductance channel can strongly modulate that of the BK_Ca_ channel [107]. 

### 4.4. Future Perspectives

#### 4.4.1. Limitation of the Usage of Chondrocyte Cell Line

Within mature mammalian articular joints, the chondrocyte exists and functions as a single, relatively isolated cell. At present most research groups obtain these preparations (single chondrocytes) using (i) immortalized chondrocyte cell lines such as TC28a2, OUMS-27, SW-1353, (ii) chondrogenic progenitor cell line (ATDC5) and (iii) primary cultured chondrocytes that enzymatically dispersed from cartilage tissues. Such immortalized cell lines are easy to handle and maintain chondrocytic phenotype, e.g., Col2a1 and So×9 expression, but at the same time they lose some chondrocytic features such as NO production, expression of genes involved in matrix synthesis and turnover [108,109,110]. We recognize that it is needed to validate the findings obtained with chondrocyte cell lines by using primary cultured chondrocytes or cartilage tissues. 

#### 4.4.2. The Chondron vs. the Chondrocyte

However, there is a limitation on using primary chondrocytes cultured in 2-D conditions. The environment of chondrocytes in 2-D culture is quite different from that within cartilage tissues. This difference is a reason why chondrocytes dedifferentiated after enzymic dissociation. It is important to recall and acknowledge that although this experimental paradigm yields useful results, for translational applications this approach may be limiting. 

In the articular joint, the functional unit is the chondrocyte together with its immediate pericellular coating. This combination has been denoted ‘the chondron’ [111]. Both classical work [112] and relatively recent data sets [113,114] have established that this pericellular matrix provides a dense coating that is separated from the chondrocyte surface. This results in the chondrocyte being surrounded by a significant diffusion barrier which includes localized highly charged residues. This ‘surface charge’ would be expected to significantly alter closely apposed ion channel and transporter function and also to contribute a significant component to the overall transmembrane potential. In addition, the pericellular matrix of the chondron is involved in transduction of applied mechanical forces and modulation of biochemical signaling pathways [115]. Recent studies [10,65,115] successfully utilized in situ Ca^2+^ imaging methods where chondrocytes in cartilage tissue were loaded with Ca^2+^ indicators and dynamic Ca^2+^ signaling were observed using a confocal microscope. 

#### 4.4.3. Extracellular Matrix Interaction with Ion Channels

Although our focus in this review, and in our experimental programs, is on ion channels in single isolated chondrocytes; we recognize the need to study the physiology of the chondrocyte in a scalable fashion by also taking account of research that relates the composition and dynamics of the extracellular matrix to both the chondron and the chondrocyte. This integrative approach [116] is necessary to relate electrophysiological findings to important elements of collagen and joint lubricant synthesis and secretion. These essential processes are energy consuming, and for that reason, overall changes in articular joint metabolism in health and in progressive disease [117] need to be carefully considered to effectively address the key question, “What have we learned about molecular transport in articular cartilage in the last 50 years?” [118]. Progress towards understanding chondrocyte physiology, the progressive changes with aging, and osteoarthritis [119] is likely to require further work on the progenitor or stem cell population of chondrocytes [78,87] and may benefit from recent advances in detecting and analyzing extracellular vesicles that are released from chondrocytes and contain very important molecular signatures [120] for ion channels or intracellular signaling pathways. It also seems possible that integrating important new findings that characterize ER stress in chondrocytes with the concepts that are diagramed in Figure 6 will be informative [121].
